# It's not what you do it's the way that it's measured: quality assessment of minor ailment management in community pharmacies

**DOI:** 10.1111/ijpp.12305

**Published:** 2016-09-28

**Authors:** Jackie Inch, Terry Porteous, Vivienne Maskrey, Annie Blyth, Jackie Burr, Jennifer Cleland, David J. Wright, Richard Holland, Christine M. Bond, Margaret C. Watson

**Affiliations:** ^1^ Centre of Academic Primary Care University of Aberdeen Aberdeen UK; ^2^ Health Services Research Unit University of Aberdeen Aberdeen UK; ^3^ Faculty of Medicine and Health Norwich Medical School Norwich Research Park University of East Anglia Norwich UK; ^4^ Division of Medical and Dental Education John Simpson Chair of Medical Education University of Aberdeen Aberdeen UK; ^5^ School of Pharmacy University of East Anglia Norwich UK; ^6^ School of Medicine Health Policy Practice University of East Anglia Norwich UK

**Keywords:** community pharmacy services, health care, non‐prescription medicines, quality indicators, self‐care

## Abstract

**Background:**

Effective management of minor ailments in community pharmacies could reduce the burden on alternative high‐cost services (general practices, Emergency Departments). Evidence is needed regarding the appropriateness of management of these conditions in community pharmacies.

**Objective:**

To explore the appropriateness of minor ailment management in community pharmacies.

**Setting:**

Prospective, observational study of simulated patient (SP) visits to community pharmacies in Grampian (Scotland) and East Anglia (England).

**Method:**

Eighteen pharmacies (nine per centre) were recruited within a 25‐mile radius of Aberdeen or Norwich. Consultations for four minor ailments were evaluated: back pain; vomiting/diarrhoea; sore throat; and eye discomfort. Each pharmacy received one SP visit per ailment (four visits/pharmacy; 72 visits total). Visits were audio‐recorded and SPs completed a data collection form immediately after each visit.

**Primary Outcome Measure:**

Each SP consultation was assessed for appropriateness against product licence, practice guidelines and study‐specific consensus standards developed by a multi‐disciplinary consensus panel.

**Results:**

Evaluable data were available for 68/72 (94.4%) visits. Most (96%) visits resulted in the sale of a product; advice alone was the outcome of three visits. All product sales complied with the product licence, 52 (76%) visits complied with practice guidelines and seven visits achieved a ‘basic’ standard according to the consensus standard.

**Conclusion:**

Appropriateness of care varied according to the standard used. Pharmacy‐specific quality standards are needed which are realistic and relevant to the pharmacy context and which reflect legal and clinical guidelines to promote the safe and effective management of minor ailments in this setting.

## Introduction

Emergency Departments (EDs) and general practices are overwhelmed by demand, due in part to patients presenting with conditions suitable for management by other service providers, including community pharmacists and their teams.^1^, ^2^ Recent estimates suggest that 5% of ED visits and 13% of general practice visits in the UK are for minor ailments that could have been managed in community pharmacies.^3^, ^4^ At the same time, there is evidence from academic^5^, ^6^ and commercial investigations^7^ of low‐quality management of these consultations in community pharmacies involving suboptimal advice or inappropriate sales. These findings are of concern at a time when there is increasing interest in promoting the advisory role of the community pharmacist in addition to the technical supply function.^8^

This study was part of the 2‐year MINA research programme,^4^ which derived evidence to inform the future delivery of minor ailment services in community pharmacies in the UK.

### Aim

The aim of this study was to explore the appropriateness (process and outcome) of minor ailment management in community pharmacies using simulated patients (SPs).

## Method

### Design and setting

A prospective, observational design was used and included community pharmacies in Grampian (Scotland) and East Anglia (England). SP visits tested the management of four minor ailments.

### Recruitment of community pharmacies

Eighteen pharmacies were required (nine each from Grampian and East Anglia), to give a wide variation in terms of type (independent, multiple) and location (urban, rural). All community pharmacies within a 25‐mile radius of the main city in each centre (Aberdeen (*n* = 35), Norwich (*n* = 30)) were identified from Health Board (Scotland) and local lists and study invitation packs were mailed. Pharmacies were excluded if they had participated in earlier phases of the MINA programme.^4^ Interested pharmacies were stratified by type and location and randomly selected to generate the sample. Signed informed consent was sought from the lead pharmacist within each participating pharmacy. Non‐pharmacist staff could opt out of participation if preferred.

### Scenario development

The four minor ailments that occur with the highest frequency in EDs and general practices^3^, ^4^, ^9^ were chosen for scenario development; they were back pain, vomiting and diarrhoea, sore throat and eye discomfort. A standardised scenario for each minor ailment (Table 1) was developed for the SP visits by a 28‐person multi‐disciplinary consensus panel^4^ (see later). Each scenario was derived from actual consultations which occurred during an earlier phase of the MINA programme.^9^

**Table 1:** Simulated patient scenario

Scenario 1	Scenario 2	Scenario 3	Scenario 4
Back pain	Sore throat	Vomiting & diarrhoea	Eye discomfort
Presentation: I need something for my back (female)	Presentation: I need something for a sore throat (male)	Presentation: I need something for vomiting and diarrhoea (female)	Presentation: I need something for my eye (female)
Symptoms, If asked; Pain in her lower back The pain began this morning when she bent over to pick up her trousers The pain is on both sides of her lower back, and has continued on and off since, when bending and coughing No other symptoms; feels well otherwise	Symptoms, If asked; Had sore throat for four days Yellow/green coloured phlegm Not able to eat anything, but trying to drink Also has headache Feels a bit hot	Symptoms, If asked; Had vomiting and diarrhoea for two days Not able to eat anything, but drinking Don't have a temperature No blood in stools	Symptoms, If asked; Discomfort in eye No visual disturbance Awoke today with a red, crusty eye, but it seems to have improved a bit No idea what could have caused this No other symptoms; otherwise well
History, if asked; 38 years old Doesn't usually have a problem back No previous trauma No sudden weight loss No relevant lifestyle issues Current job doesn't involve physical work Nothing alleviates or worsens the symptoms No other medical conditions	History, if asked; 61 year old Doesn't smoke Gets quite a lot of sore throats Doesn't have any chronic respiratory illnesses, like asthma, sinusitis, bronchitis, COPD or emphysema	History, if asked; 55 year old Son was vomiting at the weekend, but nobody else in the family has it Doesn't get this sort of thing often Hasn't been abroad recently No other medical conditions	History, if asked; In her sixties No previous eye problems Nobody else in the household has got it Doesn't wear contact lenses No other medical conditions
Treatment, if asked; Not using any medicines No known drug allergies No action taken No simple analgesia at home	Treatment, if asked; Not using any medicines No known drug allergies Has taken paracetamol for headache and sucking cough sweets to ease throat		Treatment, if asked; Not using any medicines No known drug allergies No action taken

### Simulated patients and consultations

Ten SPs (one per scenario plus one reserve per centre) were recruited from existing SP groups in the Medical Schools at the Universities of Aberdeen and East Anglia. A 4‐h training session, informed by previous studies,^10^ was delivered at each centre. Training focused on delivering a standardised performance while responding naturally to pharmacy personnel questions.^11^ Each SP was trained to perform one scenario and wore a hidden microphone to digitally record their consultations. To minimise the risk of detection and any resulting Hawthorne effect,^12^ pharmacy staff were blind to the timing and number of SP visits, as well as the ailments that would be tested. The order of SP visits to pharmacies was randomised. Participating pharmacists and pharmacy assistants wore study badges throughout the study period to ensure that SPs did not consult non‐participating staff. SPs distinguished pharmacy staff from pharmacists by uniforms worn and company name badges. Each pharmacy was supplied with reply‐paid postcards to complete (date/time/visit details) and return to the research team, if they suspected receiving an SP visit. Participating pharmacies were scheduled to receive one SP visit per minor ailment, i.e. four visits per pharmacy (72 visits in total) over an 8‐week period (February–March 2013).

### Primary outcome measure

The appropriateness of the content and outcome of each SP visit was assessed against three standards:

- the summary of product characteristics (SPC) if a product was sold/supplied;
- clinical guidelines;
- and a consensus standard (comprising consultation process and outcome) (developed by the multi‐disciplinary consensus panel).

Manufacturers’ SPCs describe medicinal product properties and the conditions for their use. Existing clinical guidelines were identified for the management of the conditions presented in each of the four scenarios. These included guidelines from SIGN (Scottish Intercollegiate Guidelines Network), NICE (National Institute for Health and Clinical Excellence), NHS Inform (a national health information service in Scotland), and the Royal Pharmaceutical Society (Table 2).

**Table 2:** Medication suggested by professional guidelines

Minor ailment	Guideline	Medication suggested by guideline
Back pain	NICE Guideline CG88^31^ & NHS Inform	Regular paracetamol, if insufficient advice NSAIDS or weak opioids
Sore throat	NHS Inform & SIGN guidelines^16^	Recommend ibuprofen and paracetamol as a first line of treatment for sore throats
Vomiting and diarrhoea	NHS Inform^17^	Keep hydrated. Advocates the use of anti‐diarrhoea medication
Eye discomfort	RPS^18^	Indicates ‘marginal benefit with chloramphenicol’, but does not discourage it's use

Scenario‐specific consensus standards (which included process and outcome) were developed by the multi‐disciplinary consensus panel which comprised community pharmacists (*n* = 8), ED consultants (*n* = 4), ED nurses (*n* = 4), GPs (*n* = 4), practice nurses (*n* = 4) and lay members (*n* = 4) from Scotland, England, Northern Ireland and Wales. Panel members operated independently and the process was conducted by email. An outline scenario was developed for each of the four minor ailments which included a list of possible consultation components in terms of content (information gathering/provision), including questions from the aide memoire known as ‘WWHAM’^13^ (Who is it for?; What are the symptoms?; How long have the symptoms been present?; Any medication tried already?; what Medication used currently?), and likely outcomes (product sale, advice provision). Panel members were asked to add any items that they perceived would be relevant or important to include in a pharmacy consultation for each scenario. The responses were collated and then fed back to panel members who were asked to indicate which components they considered represented ‘basic’ or ‘good’ practice standards for the management of the four conditions in a community pharmacy (Table 3) in terms of process and outcome. Consensus was defined as ≥60% agreement between panel members (i.e. ≥17/28) for both ‘basic’ or ‘good’ consultation management. Only items where the consensus threshold was met were included in the final standard. When using these standards to assess SP consultations, all components had to be delivered for the consultation to meet the consensus standard for process.

**Table 3:** Consultation components to represent ‘basic’ and ‘good’ practice as recommended by the multidisciplinary consensus panel

Scenario 1: Back pain	Scenario 2: Sore throat	Scenario 3: Vomiting & diarrhoea	Scenario: 4 Eye discomfort
**What are the symptoms?**	**What are the symptoms?**	**What are the symptoms?**	**What are the symptoms?**
**Who is the patient?**	**How long have the symptoms been present?**	**Who is the patient?**	**How long have the symptoms been present?**
**How long have the symptoms been present?**	**Are any other medications being used (for other conditions)?**	**How long have the symptoms been present?**	**Is this patient a contact lens wearer?**
**Are any other medications being used (for other conditions)?**	**Advise re. symptoms and action if symptoms continue**	**Are any other medications being used (for other conditions)?**	**Ensure advice re no contact lenses until condition resolved and ensure good cleaning of lenses if not disposable**
**Does the patient have other medical conditions?**	**Timescale defined (no improvement after × period referral advised)**	**Does the patient have other medical conditions?**	Who is the patient?
**Dosage instructions (if medication has been recommended)**	Who is the patient?	**Has the patient stopped taking oral fluids?**	**Dosage instructions (if medication has been recommended)**
**Advise re. symptoms and action if symptoms continue**	How old is the patient?	**Advise re hydration/fluids**	**Advise re. symptoms and action if symptoms continue**
Does the patient have any other symptoms?	Has any action been taken?	**Advise re. symptoms and action if symptoms continue**	**Timescale defined (no improvement after** ×** period referral advised)**
Has any action been taken?	Does the patient have other medical conditions?	**Dosage instructions (if medication has been recommended)**	Has any action been taken?
Does anything alleviate or worsen symptoms	Is there any associated temperature?	Has any action been taken?	Are any other medications being used (for other conditions)?
Check understanding	Does the patient have a history of chronic or recurrent sinusitis?	Have they tried non‐prescribed over the counter, herbal or home remedies?	Does the patient have other medical conditions?
Ask if already have simple analgesia at home?	Does the patient have a chronic respiratory illness?	Any other members of the household affected?	Have they had previous eye problems e.g. scleritis/iritis?
Relevant product information (if a product recommended)	Does the patient have any other symptoms?	Does the patient have any other symptoms?	Ensure there is no pain from within the eye, and that there exists no disturbances from bright lights. (Rule out iritis)
If anti‐inflammatories suggested check contra indications	Check understanding	Has there been any recent travel e.g. Overseas?	Is there a history of possible foreign body/eye abrasion
Encourage early mobilisation for muscular back pain	Relevant product information (if a product recommended)	Is there any blood present in the stools (faeces)?	Are there any other members of the household affected?
Refer to GP ‘if symptoms persist’	Dosage instructions (if medication has been recommended)	Do you have a temperature?	Is the patient using the children's medication?
	Ask if there are any questions	Blood in stools ‐ if so refer to GP	Advise about actions to prevent spread of infection
		Are there any clinical signs of dehydration? ‐ reduced urine output, tachycardia?	Advice about hygiene within the household and not re‐infecting family members (cross infection)
		Check understanding	Check understanding
		Timescale defined (no improvement after × period referral advised)	Advice about self limiting nature of viral or bacterial conjunctivitis in otherwise healthy individuals
		Advise about actions to prevent spread of infection	Relevant product information (if a product recommended)
		Advise about handwashing/food contact etc.	

Bold text represents ‘basic’ consultation requirements of the Multi‐disciplinary Consensus Panel. Normal print represents additional requirements to reflect a ‘good’ consultation.

### Data collection

There were two components to the data collection; the digital recordings of the consultation (see above) and a data collection form completed after every visit by the SP. This included:

- Staff involved (pharmacist/counter staff)
- Questions asked
- Outcome of visit (medication/advice supplied)
- Perceived professionalism of staff involved (5‐point scale)
- Overall satisfaction with visit (5‐point scale)
- Number of other customers on premises (as a marker of busyness)
- Duration of the visit (from digital recorder)

### Assessment of the management of minor ailments presented during SP visits

Three researchers (JI, JC and JB) independently validated the data from the data collection forms against digital recordings for quality assurance purposes. Each researcher listened to each digital recording and compared their interpretations with the SP data collection forms. There were few discrepancies and consensus was defined as when two out of three researchers were in agreement.

### Data management and analysis

All data were entered into SPSS version 20.^14^ Independent accuracy checks were performed on 10% of the data. The results are presented as descriptive statistics.

### Ethical approval

Ethical approval was granted by the College of Life Sciences and Medicine Ethics Review Board, University of Aberdeen.

## Results

Twenty pharmacies (31% (20/65)) consented to participate; 18 were selected (independent (6), small chain (4) and large chain (8)). Pharmacies were located in urban (5) and suburban (7) areas and small towns (6).

All planned SP visits (*n* = 72) were completed. Two pharmacies reported ‘suspected’ SP visits, one of which corresponded to an actual visit; data from this visit were excluded from analysis. Digital data were unavailable for three visits (due to recording problems), so these were also excluded from the analysis. Evaluable data were available for 68/72 (94.4%) visits. Most (96% *n* = 65) visits resulted in the sale of a product; advice alone was the outcome of three visits. One‐third of visits involved interaction with a pharmacist only. Most visits were conducted when the pharmacies had <3 customers and most SPs waited between 1 and 5 min for their consultation. Three visits lasted more than five minutes (Table 4).

**Table 4:** Summary of consultation characteristics (%*n* (derived from data collection form)

Characteristic	% (*n*)	% (*n*)	% (*n*)
Members of staff involved in consultation	Pharmacist	Non‐pharmacist	Unknown
	38.9 (28)	66.7 (48)	18.0 (13)
Duration of consultation (minutes)	≤1	2–5	>5
	33.3 (24)	58.5 (45)	4.2 (3)
Duration of wait prior to consultation (minutes)	≤1	**–**	>5
	72.2 (52)	26.4 (19)	1.4 (1)
Busyness of pharmacy (number of customers waiting at time of SP consultation)	0	1–2	≥3

### Appropriateness of consultation process and outcome

**Figure 1:**
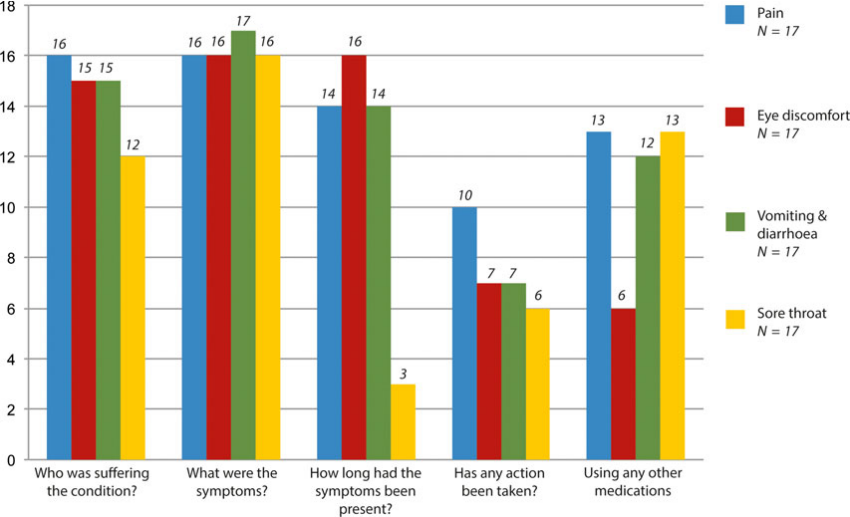
Content of simulated patient consultations (in relation to WWHAM REF protocol) (%(*n*): by scenario.

All product sales (outcome) complied with the product licence, 52 (76%) visits complied with practice guidelines and seven visits achieved a ‘basic’ standard according to the consensus standard for process (Tables 5 and 6). No consultation achieved a ‘good’ standard of practice for process. In terms of the content of SP consultations, the extent to which WWHAM^15^ components were elicited varied substantially across scenarios (Figure 1).

**Table 5:** Assessment of SP consultation outcomes against the summary of product characteristics, guidelines and consensus list proposed outcome

	Scenario			
Appropriateness of outcome compared with:	Back pain (*N* = 17) % (*n*)	Sore throat (*N* = 17) % (*n*)	Vomiting & diarrhoea (*N* = 17) % (*n*)	Eye discomfort (*N* = 17) % (*n*)
Consensus list proposed outcome	100 (17)	100 (17)	15.8 (1)	15.8 (1)
Summaries of product characteristics	100 (17)	100 (17)	100 (17)	100 (17)
NICE Guideline^30^	100 (17)			
NHS Inform (Scotland)^17^	100 (17)	82.3 (14)	58.8 (10)	
SIGN guideline (117), April 2010^16^		0 (0)		
Royal pharmaceutical society reference guide^18^				64.7 (11)
Outcome of Consultation				
Appropriate outcome	100 (17)	100 (17)	5.8 (1)	5.8 (1)
Product sold	94.1 (16)	100 (17)	94.1 (16)	94.1 (16)
	Ibuprofen + Curaheat pad (2)	Difflam/Covonia/Chloraseptic throat sprays × 7	Dioralyte + Loperamide × 2	Brolene × 1
	Ibuprofen × 12	Dry cough linctus × 3	Pepto‐Bismol × 4	Tubilux × 1
	Ibuprofen + Paracetamol × 2	Strepsils/Tyrozets lozenges × 4	Dioralyte + Buccastem × 1	Chloramphenical × 11
		Strefen × 1	Loperamide × 8	Optrex eye drops × 2
		Strepsils + Ultrachloraseptic throat spray × 1	Motilium × 1	Golden eye ointment × 1
		Tyrozets + Benylin dry cough × 1		
No sale	5.8 (1)	0	5.8 (1)	5.8 (1)

**Table 6:** Assessment of SP consultation content against the consensus list

	Back pain *N* = 17	Eye discomfort *N* = 17	Vomiting and diarrhoea *N* = 17	Sore throat *N* = 17
Consultation content (Basic)	*n* (sp)	*n* (sp)	*n* (sp)	*n* (sp)
**What are the symptoms?**	16 (8)	16 (5)	17 (12)	16 (5)
**Who is the patient?**	16 (16)	–	15	–
**How long have the symptoms been present?**	14 (9)	16 (10)	14 (1)	3 (2)
**Are any other medications being used (for other conditions)?**	13	–	12	13
Does the patient have other medical conditions?	9	–	2	–
Advise re. symptoms and action if symptoms continue	7	9	9	2
Is this patient a contact lens wearer?	–	5	–	–
Ensure advice re no contact lenses until condition resolved and ensure good cleaning of lenses if not disposable	–	a0	–	–
Has the patient stopped taking oral fluids?	–	–	0	–
Advise re hydration/fluids	–	–	15 (1)	–
Dosage instructions (if medication has been recommended)	14	12	6	–
Timescale defined (no improvement after a specified period referral advised)	–	6	–	2
General professionalism (SP rating)	*n*	*n*	*n*	*n*
Exceptional interaction with member of staff	7	7	5	6
Member of Staff helpful, polite and competent	10	8	12	10
Non‐professional approach, lacked confidence	0	2	0	1
Satisfaction with Consultation (SP rating)	% (*n*)	% (*n*)	% (*n*)	% (*n*)
Satisfied	94.1 (16)	64.7 (11)	94.1 (16)	100.0 (17)
Not satisfied including uncertain	5.9 (1)	35.3 (6)	5.9 (1)	0

(SP) Indicates where the SP provided unsolicited information.

a Questions/advice that was conditional on answers of other questions. Bold items represent WWHAM^15^ items.

*Back pain (n =* 17): In terms of products sold, all the back pain scenario visits resulted in appropriate outcomes when assessed against both the clinical guidelines and consensus panel standard (Table 5).

*Sore throat* (*n* = 17): The SIGN guideline^16^ states that there is no good quality evidence for non‐prescription throat sprays, lozenges or gargles. No visit complied with the clinical guidelines because they all resulted in the sale of at least one of these products. In contrast, however, all sales complied with the ‘basic’ consensus standard in terms of outcome, i.e. products sold.

*Vomiting and diarrhoea* (*n* = 17): Ten consultations complied with NHS Inform guidelines^17^ which advocate the use of an anti‐diarrhoeal product. Six SP visits resulted in the sale of anti‐nausea or anti‐emetic products, the use of which was not supported by the guideline. Only one consultation fulfilled the basic consensus standard for outcome which advocated advice only.

*Eye discomfort* (*n* = 17): Eleven consultations resulted in the sale of chloramphenicol products (suggested as having marginal benefit in the RPSGB guideline^18^). One visit resulted in advice only and was therefore compliant with the ‘basic’ outcome standard advocated by the consensus panel. Five other consultations resulted in other eye products being sold.

### General professionalism and satisfaction

SPs rated the visits highly in terms of the general professionalism of pharmacy staff involved in their consultation (Table 6). High levels of satisfaction (very satisfied/satisfied) were also reported. Of the eight visits where the SP was not satisfied, six involved the same SP.

## Discussion

### Summary

The appropriateness of the SP visits in terms of process and outcome varied according to the standard used. All SP consultations complied with the SPCs. The majority (*n* = 76%) of visits also complied with clinical guidelines. Few consultations reflected the standards defined by the consensus panel, particularly in terms of information gathering.

### Strengths and limitations

Discrepancies have previously been shown to exist between self‐reported and actual recorded data,^19^ but in this study, the digital recordings provided objective data for analysis. Non‐verbal communication is an important component of healthcare consultations,^20^ but was not assessed in this study. Duplicate, independent data extraction and assessment reduced the risk of assessor bias.

The inclusion of different types and locations of pharmacies in two countries with different pharmacy service contracts increased the generalisability of the results. Pharmacies self‐selected to participate in the study. No data were collected from the non‐participants. It is possible that the participating pharmacies may have had higher levels of appropriate practice compared with non‐participants and as such, the results may overestimate appropriate practice across all pharmacies. Furthermore, the inclusion of pharmacies within a 25‐mile radius of the study centre may not be comparable with national data due to the omission of rural pharmacies. Female customers are higher users of pharmacies than males, therefore, to maximise face validity, most of the SPs in this study were female. However, there is evidence to suggest that patient gender can influence the type of information given by pharmacists during counselling.^21^ Pharmacist/counter assistant gender may also influence communication during consultations^22^, but pharmacy personnel gender was not recorded in this current study.

### General discussion

This study clearly demonstrates that the choice of standard impacts substantially on the assessment of appropriateness. To the best of our knowledge, this has not been reported previously and is a novel finding of our study. Some of the clinical guidelines and consensus standards (for outcome) conflicted with the broader SPC indications. It is unclear to what extent the clinical guidelines used in this study are referred to routinely in community pharmacy practice. No condition‐specific clinical guidelines were identified that had been developed for use in community pharmacies; only product‐specific guidelines were identified, e.g. chloramphenicol.^18^ Our study highlights a need for clinical guidance to be tailored for use by community pharmacists and their teams and which does not conflict with other accepted guidance. The current lack of standardised tools will not only contribute to a lack of consistency between pharmacists and other health care providers, but may lead to variation in clinical practice and appropriateness of care that is so often reported in the wider international literature.^6^ In the UK, the General Pharmaceutical Council is developing standards for the sale or supply of Pharmacy medicines,^23^ but the content is as yet unspecified and unlikely to be condition‐specific.

The multidisciplinary consensus panel generated ‘basic’ and ‘good’ standards for this study. Although the panel included eight community pharmacists, the components of some standards could be considered unrealistic for the management of minor ailments in the community pharmacy context. For example, the vomiting and diarrhoea standard included eliciting whether there were any clinical signs of dehydration including ‘reduced urine output, tachycardia’ which lack face validity in a community pharmacy setting. Other components of the ‘good’ standard were also problematic. For example, few ‘sore eye’ scenario consultations included elicitation of whether the SP was using other medication; there are, however, no major drug interactions associated with the topical use of chloramphenicol which calls into question the validity of this component. These examples demonstrate not only the challenge of measuring appropriateness of healthcare management in this setting but also the potential lack of validity of using standard generic guidance, e.g. WWHAM, to assess a range of conditions when all items may not be relevant.

### Consultation process

While community pharmacists and staff in this study asked questions (information gathering) and gave advice in the majority of consultations, some items central to safe supply were asked infrequently. For example, while SPs were often asked about current medication, they were rarely asked about co‐morbidities. Similarly, information about how to use medicines was commonly provided, but advice about what to do should symptoms continue was rarely given. Consultations that focus on directions for medication use and dose, rather than on side effects and adverse events, were also identified in a review of counselling practices on prescription medicines in community pharmacies.^24^ However, the extent and type of communication between SPs and pharmacists in our study was similar to other studies of OTC medicines in the UK.^11^, ^25^, ^26^

### Comparison with existing literature

Effective consultations between pharmacy staff and their patients/customers are crucial to ensuring appropriate medication use and desired patient outcomes in terms of increased patient knowledge, adherence and/or decreased medication errors.^6^, ^27^, ^28^ Hence, training in information gathering and provision (consultation skills) is now a core part of the pharmacy undergraduate and post‐qualification training programmes.^29^ The relatively recent Medication Related Consultation Framework (MRCF)^30^ is promoted by the Royal Pharmaceutical Society in the UK as a tool for pharmacists to develop their consultation skills in general. However, elements of the framework lack face validity in relation to the management of over‐the‐counter consultations, e.g. confirmation of patient's identify, documentation of full medical history. Furthermore, most consultations for minor ailments or for OTC medicine requests are dealt with by medicine counter assistants for whom there is no organisation currently responsible (in the UK at least) for providing ongoing training (although it is the responsibility of the pharmacist‐in‐charge to ensure that staff are competent to deliver their tasks). Indeed, in some countries, e.g. Australia, there is no requirement for support staff to have undertaken any training prior to their involvement in selling or recommending medicines (Pers. Commun, L. Seubert, 2014). The need for tighter regulatory control for pharmacy support staff is paramount, as is the provision of effective and ongoing training for these vital members of the pharmacy workforce.

### Research implications

Our study highlights the difficulty of measuring the appropriateness of minor ailment management in the community pharmacy setting. Quality standards are needed by which practice can be consistently, fairly and accurately assessed. The effect on practice of applying such standards also needs to be evaluated. Effective training methods are needed to enhance consultation management in general and communication skills in particular, e.g. information gathering. While there has been minimal evaluation of this to date, there is evidence to suggest that communication behaviour can be enhanced by training.^5^

## Conclusion

The management of minor ailments was appropriate when assessed by two of the three standards used. Lower rates of appropriateness were achieved with the more aspirational consensus standards. There is a need for pharmacists and their staff to enhance their consultation skills not only through improved communication performance, but also by expanding their knowledge of health conditions. This would maximise the effectiveness of the management of minor ailments in the community pharmacy setting. We suggest that the development of quality standards for the management of such conditions is one strategy to improve practice and achieve safe and effective patient care. The use of formal standards would also provide pharmacists and their staff the criteria against which they could expect to be assessed.

## Declarations

### Conflict of interest

The authors declare that they have no conflicts of interest to disclose.

### Funding

This independent research was supported by the Pharmacy Practice Research Trust (PPRT) which merged with the Pharmaceutical Trust for Educational and Charitable Objects in December 2012 to form Pharmacy Research UK (PRUK). The views expressed in this publication are those of the authors and not necessarily those of the PPRT or of PRUK.

### Authors’ contributions

JI and JB contributed to acquisition of data, analysis and interpretation of data. TP contributed to conception and study design and critically revising the final document. VM and AB contributed towards study design and acquisition of data. JC contributed towards conception, design, interpretation of data and critically revising the final document. DJW and RH contributed to conception, design and interpretation of data. MCW and CMB contributed to conception, design and analysis, interpretation of data, drafting, critically revising and final approval of paper and of report.
